# Task-Dependent Interaction between Parietal and Contralateral Primary Motor Cortex during Explicit versus Implicit Motor Imagery

**DOI:** 10.1371/journal.pone.0037850

**Published:** 2012-05-31

**Authors:** Florent Lebon, Martin Lotze, Cathy M. Stinear, Winston D. Byblow

**Affiliations:** 1 Neurology Research Group, Department of Medicine, University of Auckland, Auckland, New Zealand; 2 Functional Imaging, Diagnostic Radiology and Neuroradiology, University of Greifswald, Greifswald, Germany; 3 Centre for Brain Research, University of Auckland, Auckland, New Zealand; 4 Movement Neuroscience Laboratory, Department of Sport and Exercise Science, University of Auckland, Auckland, New Zealand; Katholieke Universiteit Leuven, Belgium

## Abstract

Both mental rotation (MR) and motor imagery (MI) involve an internalization of movement within motor and parietal cortex. Transcranial magnetic stimulation (TMS) techniques allow for a task-dependent investigation of the interhemispheric interaction between these areas. We used image-guided dual-coil TMS to investigate interactions between right inferior parietal lobe (rIPL) and left primary motor cortex (M1) in 11 healthy participants. They performed MI (right index-thumb pinching in time with a 1 Hz metronome) or hand MR tasks, while motor evoked potentials (MEPs) were recorded from right first dorsal interosseous. At rest, rIPL conditioning 6 ms prior to M1 stimulation facilitated MEPs in all participants, whereas this facilitation was abolished during MR. While rIPL conditioning 12 ms prior to M1 stimulation had no effect on MEPs at rest, it suppressed corticomotor excitability during MI. These results support the idea that rIPL forms part of a distinct inhibitory network that may prevent unwanted movement during imagery tasks.

## Introduction

Motor imagery (MI) is the mental representation of action and is associated with processes observed during motor preparation [1]. Mental rotation of objects and hands (MR) is a higher-order visuospatial task that also involves imagery processes [2], [3]. Both MI and MR of hands involve a spatial construct of moving body parts and an internal strategy without any concomitant movement [4]. A key distinction between MI and MR is that MI is a form of explicit imagery whereas MR is a form of implicit imagery [5], [6], [7]. De Lange and colleagues [5] described the two types of motor imagery: “During explicit imagery tasks subjects are simply asked to imagine moving their effector in a particular manner […]. Implicit imagery tasks on the other hand usually employ a task that is tangential to imagery of actions […], and infer the motoric nature of the processes involved in solving the task from the behavior of the subjects”. Despite this distinction, brain imaging studies indicate that MI and MR are sustained by comparable brain activation profiles, including motor cortices [8] and the right inferior parietal lobe (rIPL). The rIPL plays an important role in predicting the sensory outcome of mentally simulated action [9]. Interestingly, parietal lesions lead to impairments in MI such as decrements in predicting the time required for completing a movement [10], [11] or an inability to prevent overt movement during MI [12]. Therefore, movement inhibition is thought to engage either right or left parietal lobe or both. Moreover, virtual lesion studies of the right parietal lobe demonstrate degraded accuracy of MI [13] and delayed responses during MR [14]. Until now, the role of the right parietal cortex in motor inhibition has not been studied in detail during imagery.

Transcranial magnetic stimulation (TMS) has been used to explore the neural mechanisms of MI and MR and can be used to assess cortico-cortical interactions. Single-pulse TMS of primary motor cortex indicates there is a muscle- and time-specific facilitation of corticomotor excitability during MI [15], [16]. However, modulation of corticomotor excitability during MR is unclear. Bode et al. [17] and Eisenegger et al. [18] reported facilitation of corticomotor excitability, whereas Wraga et al. [19] and Sauner et al. [20] did not. These differences may have been due to timing of TMS. Recently, dual-coil TMS has been used to identify functional coupling between the parietal lobe and M1 at rest. For example, Koch et al. [21] showed that conditioning right caudal intraparietal sulcus before stimulating left M1 facilitated the amplitude of the resulting motor evoked potential (MEP) recorded in right first dorsal interosseous (FDI). In contrast, conditioning right anterior intraparietal sulcus suppressed FDI MEP amplitude [22]. These studies indicate that the parietal lobe can exert both facilitatory and suppressive effects on the contralateral M1 at rest. However, nothing is yet known about the influence of right parietal lobe on left M1 during MI or MR. Based on previous findings observed during resting conditions, the present study was conducted to assess imagery task-dependent modulation of inter-hemispheric connectivity between rIPL and left M1.

In the present study, we focussed on inhibitory interactions between rIPL and left M1 during MI and MR. We hypothesized that conditioning rIPL would have a suppressive effect on left M1 corticomotor excitability during both MI and MR, indicative of a putative mechanism to prevent actual movement. Two different time windows, 6 and 12 ms ISI, were used to assess putative direct (cortico-cortical) and indirect (cortico-basal ganglia-thalamo-cortical) pathways respectively.

## Materials and Methods

### Participants

Seventeen healthy adults (8 females; mean age 24 years, range 19–46 years) took part in the study. Ethical approval for the study was granted by the University of Auckland Human Participants Ethics Committee and all participants gave written informed consent. All participants were screened using TMS and MRI safety checklists. They were all deemed right-handed with the Edinburgh Handedness Inventory [23] (mean score 68, range 54–91). Eleven participants (aged 19–46, 5 females) demonstrated the expected facilitation of MEPs at rest with rIPL conditioning and a 6 ms ISI [21], and were included in the final analysis. Edinburgh handedness scores ranged from 54–91. Data from six participants were discarded because stimulation of rIPL did not induce MEP facilitation at rest, and thus effective rIPL conditioning could not be verified using this combination of coordinate, conditioning stimulus intensity and ISI.

### Dual-coil Transcranial Magnetic Stimulation

For neuro-navigation of coil positions, T1-weighted magnetic resonance images were acquired using a 3 T Magnetom Skyra (Siemens, Erlangen, Germany). High-resolution structural images of the whole head were acquired for each participant (MPRAGE, 176 sagittal slices, repetition time = 1900 ms, echo time = 2.52 ms, flip angle 90°, voxel size 1.0×1.0×1.0 mm). These images were then co-registered with the participant using Brainsight software (Rogue Research, Montreal, Canada) and a Polaris (Northern Digital, Waterloo, Canada) infrared tracking system. The Brainsight system was used to identify and mark the positions of the TMS coils over left M1 and rIPL.

**Figure 1:**
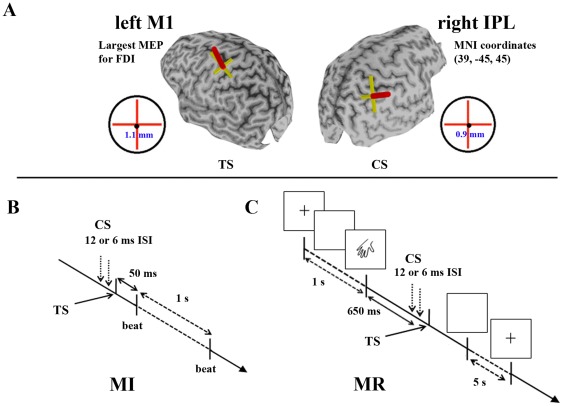
Neuro-navigated dual-coil TMS. **A.** An individual 3D rendered brain from a T1-weighted image. The cortical representation of the right FDI was marked on the left hemisphere (upper left). A site for conditioning stimulation was localized within right inferior parietal lobe at the MNI coordinate [37, −45, 46] (upper right). A target for each hemisphere was used to guide the position of the each coil. **B.** During MI, test stimulus (TS) was triggered 50 ms prior to every fifth to seventh metronome beat. **C.** During MR, TS was triggered 650 ms after the presentation of the image. During both tasks, conditioning stimulus (CS) was triggered 6 or 12 ms prior to TS.

Dual-coil TMS was delivered using 2 figure-of-eight coils (external wing diameter 8.5 and 8 cm, respectively) attached to Magstim 200 stimulators (Magstim Co., Whitland, Dyfed, UK). TMS of left M1 targeted the representation of the right FDI muscle and the coil was oriented to induce a posterior to anterior current in left M1. The optimal location to elicit MEPs in right FDI was found and marked on the 3D image of the brain, enabling consistent placement. Conditioning stimuli were delivered with a coil positioned over rIPL, oriented to induce posterior to anterior directed current in rIPL. The coordinates for rIPL stimulation were indicated in MNI space (37, −45, 46) and have good correspondence with activation peaks from a number of MI studies [24], [25], [26], [27], [28], [29] and MR studies [8], [14], [30], [31], [32]. These coordinates were anatomically located in the right inferior parietal lobule [33]. The MNI coordinate was marked on each participant's scan by warping the MNI T1 template brain to the individual's T1 volume using SPM8 (Wellcome Department of Cognitive Neuroscience, London, UK). An example of localization of both coils with respect to a 3D brain image indicating left M1 and rIPL sites is shown in Figure 1A.

Right FDI electromyography (EMG) was acquired with a Grass P511 amplifier (gain 1000), bandpass filtered (20 Hz–1 kHz), sampled at 2 kHz, and acquired using Signal v.4.08 (CED Ltd, Cambridge, England). Rest motor threshold of right FDI was determined [34]. The conditioning stimulus intensity was set at 90% of rest motor threshold, as defined by Koch et al. [21]. Test stimulus intensity was set to produce 50% of the maximal MEP amplitude at rest. The conditioning stimulus was delivered prior to test stimulus with 6 and 12 ms interstimulus intervals (ISI). Eighteen non-conditioned (NC) MEPs and 21 conditioned (C) MEPs with each ISI were collected for each task. The effect of conditioning was determined by calculating the C/NC ratio for each task. Trials were rejected if root mean squared (_RMS_EMG) exceeded 10 µV for the period 100 ms prior to TMS to ensure effects of MI and MR on MEP amplitude were not contaminated by muscle activity.

### MI and MR Tasks

The tasks (rest, MI and MR) were undertaken in a pseudo-randomized order. Within the same block of trials, conditioned and non-conditioned stimuli and no TMS trials were randomly distributed, to avoid any unwanted familiarization of additional sensory cue. During rest, participants were instructed to relax and keep their eyes open. At the beginning of each MI task, the participants physically performed three repetitions of a pinching movement with their right index and thumb in time with a 1 Hz metronome and then relaxed the hand and continued imagining the same movement in time with the metronome. Participants closed their eyes while performing MI and told to ‘imagine they were making the movement and the feeling it produced’, to reinforce the use of a kinaesthetic strategy. This is known to maximally modulate corticomotor excitability [35]. TMS was triggered 50 ms prior to every 5^th^ to 7^th^ metronome beat during MI (Figure 1B). During MI, it is known that participants use an anticipatory strategy similar to actual movement and show an increase in corticomotor excitability (MEP amplitude) 50 ms prior to the metronome beat to which they synchronize their actual or imagined movements [15], [16], [36]. After each MI block, the participants scored the vividness of their imagined movement using a 7-point Likert scale: 1 = very hard to feel, 4 = not easy/not hard, 7 = very easy to feel, 2, 3, 5 and 6 being intermediate levels.

For the MR task, images of either a left or right hand were shown in various orientations on a computer screen for 1 s. The goal was to determine if a left or right hand was shown but without verbalizing the answer to avoid any activation of motor areas involved in response (manual or speech) production. TMS was delivered 650 ms after the presentation of each image (Figure 1C), to be sure that the participants were engaged in the mental rotation tasks. Indeed, Ganis et al. [37] showed that response times (RTs) were slower when TMS was delivered at 650 ms over M1 but not at 400 ms after stimulus onset. Eighty pictures (half left; 25% with 0°, 42% with 90°, 25% with 180° and 8% with 270° hand orientation from vertical) were presented in a randomized order. After each MR block, participants scored the level of concentration and the easiness of the task using a 7-point Likert scale: 1 = no concentration/easy, 7 = high concentration/difficult. After the TMS investigation, MR accuracy was tested by presenting 24 images of hands (half left) and the participant verbally stated whether a left or right hand was presented.

### Statistical analysis

SPSS (V20, IBM Corporation, USA) was used for statistical analysis. NC MEP_AMP_ was analyzed using a 3 task (rest, MI and MR) repeated measure (RM) ANOVA. NC MEP_AMP_ during MI and MR was then normalized to rest data. Based on the modulation of normalized NC MEP_AMP_, participants were distributed into 2 groups *a posteriori*: they were considered Imagers (N = 6) when normalized NC MEP_AMP_ was greater during MI than MR, and Rotators (N = 5) when the opposite pattern was observed (see Results below). To confirm the effect of group on normalized NC MEP_AMP_, a between-subject factor of group was added to the RM-ANOVA, with one sample t-tests for post hoc analyses.

In order to explore the effect of rIPL conditioning on C/NC MEP_AMP_, we conducted a 3 task (rest, MI and MR) and 2 isi (6 and 12 ms) RM-ANOVA with a between-subject factor of group. Facilitation and suppression effects of conditioning were tested with one sample t-tests.

Pre-trigger _RMS_EMG was analyzed using a 3 task (Rest, MI, MR)×3 stimulation (NC, 6 and 12 ms) RM-ANOVA.

MI and MR self-estimation ratings were compared between blocks using repeated measure ANOVA. MR accuracy scores were tested with a Kolmogorov-Smirnov one sample test using normal distribution to test group homogeneity.

Data are presented as mean ± SD and statistical significance was α = 0.05 after correction for multiple comparisons. Greenhouse-Geisser correction was used when sphericity was violated.

## Results

### Corticomotor excitability

**Figure 2:**
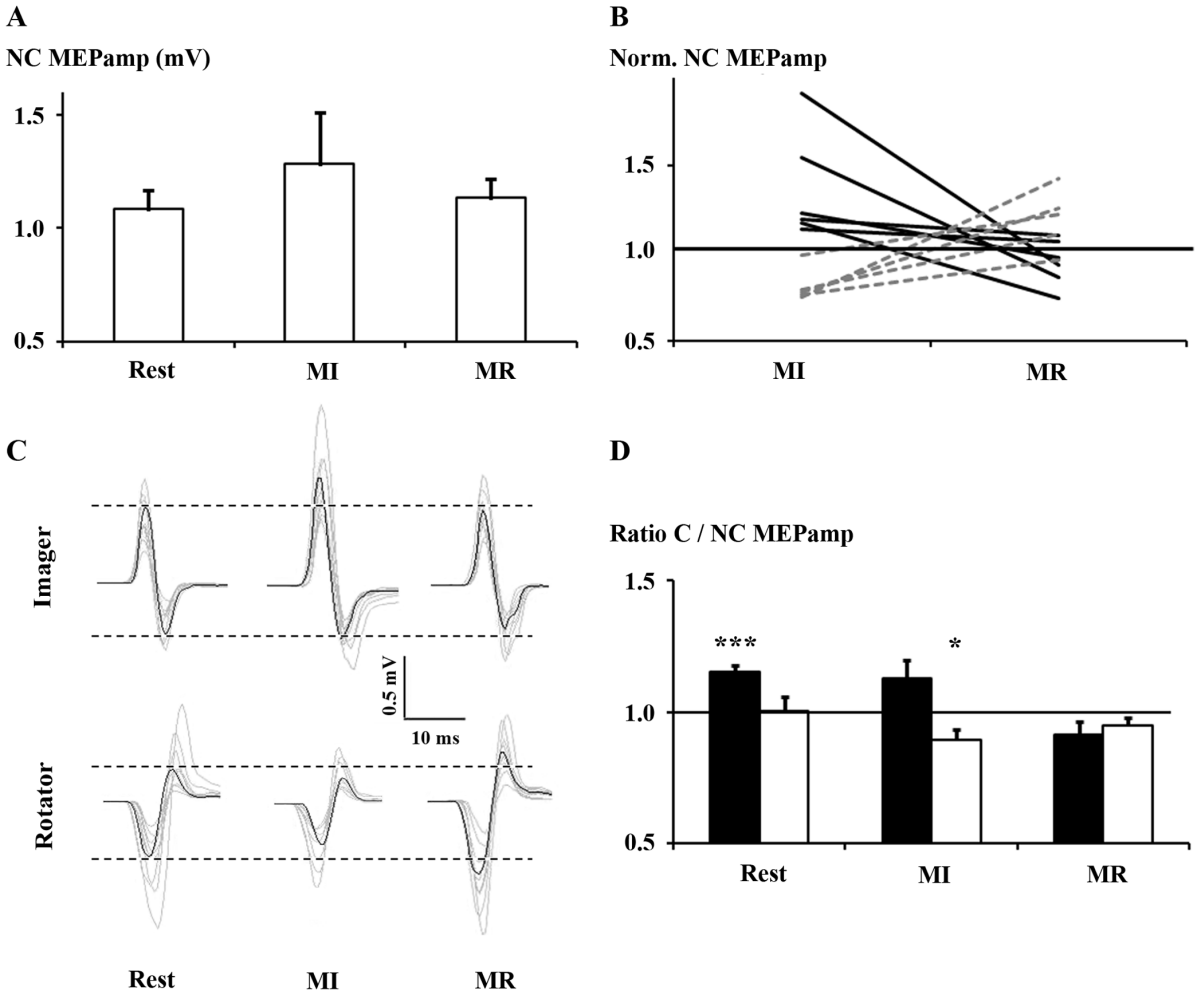
Motor evoked potentials. **A.** Mean NC MEP_AMP_ during rest, MI and MR. **B.** Two groups were distinguished according to the difference between normalized NC MEP_AMP_ during MI versus MR. Imagers (N = 6, black lines) preferentially facilitated MEPs during MI, relative to MR. Rotators (N = 5, dashed grey lines) preferentially facilitated MEPs during MR, relative to MI. **C.** Motor evoked potentials of a typical Imager and a typical Rotator. Black and grey lines represent average and individual MEPs, respectively. **D.** C/NC MEP_AMP_ ratio. With a 6 ms ISI, the ratio at rest (1.15±0.09) was greater than 1, indicating facilitation. With a 12 ms ISI, the ratio during MI was less than 1 (0.90±0.12), indicating suppression. MI = motor imagery, MR = mental rotation. * p<0.05 and *** p<0.001 for one sample t-test. Error bars indicate 1 S.E.

As intended, the test stimulus produced a NC MEP_AMP_ that ranged from 38–80% of the individual MEP_MAX_ (average 58.9%). NC MEP_AMP_ was similar between rest (1.09±0.27 mV), MI (1.29±0.75 mV) and MR (1.14±0.29 mV, Figure 2A). MEP_AMP_ variability (SD) was greater during MI than at rest and during MR, that could explain the absence of task effect (F_2,20_ = 0.975, *P* = 0.354, corrected). Indeed, NC MEP_AMP_ was modulated in two distinct patterns (Figure 2B). Two groups of participants were identified *a posteriori* based on the relative degree of FDI NC MEP_AMP_ facilitation during task performance (Figure 2C). We termed these groups Imagers and Rotators. Imagers (N = 6) exhibited greater normalized NC MEP_AMP_ during MI than MR, while Rotators (N = 5) exhibited the opposite pattern. The division was confirmed with an ANOVA of normalized NC MEP amplitude with factors group (Imagers, Rotators) and task (MI, MR). As expected there was an interaction between task and group (F_2,9_ = 19.571, *P* = 0.002, Table 1). One sample t-tests indicated that for Imagers normalized NC MEP_AMP_ was greater than rest during MI but was not different than rest during MR. For Rotators, normalized NC MEP_AMP_ was facilitated during MR and suppressed during MI (Table 1).

**Table 1:** One-sample t-tests and 95% CI of normalized NC MEP_AMP_ and C/NC MEP_AMP_ ratio.

Task	Participants	NC MEP_AMP_ (SD)	t	P-value	95% CI
MI	Imagers	1.36 (0.30)	2.91	0.016	0.043, 0.683
	Rotators	0.81 (0.10)	−4.17	0.007	−0.310, −0.062
MR	Imagers	0.95 (0.13)	0.972	0.376	−0.194, 0.088
	Rotators	1.19 (0.17)	2.50	0.034	−0.021, 0.413

ISI	Task	C/NC (SD)	t	P-value	95% CI
6 ms	Rest	1.15 (0.09)	5.49	<0.001	0.091, 0.216
	MI	1.13 (0.24)	1.79	0.104	−0.031, 0.288
	MR	0.92 (0.11)	−1.68	0.123	−0.192, 0.027
12 ms	Rest	1.00 (0.18)	0.07	0.948	−0.117, 0.125
	MI	0.90 (0.12)	−2.76	0.020	−0.187, −0.020
	MR	0.95 (0.11)	−1.62	0.136	−0.123, 0.019

MI = motor imagery, MR = mental rotation, C = conditioned, NC = non conditioned, MEP_AMP_ = mean amplitude of motor evoked potentials, ISI = interstimulus interval, SD = standard deviation, CI = confidence interval.

### rIPL – left M1 connectivity

The ANOVA of C/NC MEP_AMP_ revealed an interaction between task and isi (F_2,18_ = 3.897, *P* = 0.039). There was no main effect or interactions with group (Imagers and Rotators, all *P*>0.05). The task and isi interaction arose because with a 6 ms ISI, conditioning facilitated MEPs at Rest only, whereas it suppressed MEPs with a 12 ms ISI during MI only (Table 1, Figure 2D). With a 6 ms ISI one sample t-tests indicated that conditioned MEPs were facilitated at rest (1.15±0.09, *P*<0.001) but not during MI (1.13±0.24) or MR (0.92±0.11, both *P*>0.104). With a 12 ms ISI, conditioned MEPs were suppressed during MI (0.90±0.12, *P* = 0.020) but not at rest (1.00±0.18) or during MR (0.95±0.11, both P>0.136). Pre trigger _RMS_EMG was similar between tasks and stimuli with no main effects or interactions (all P>0.142) and all mean _RMS_EMG levels falling between 7.9–8.1 µV.

### MI and MR performance assessment and self-estimation

Estimations of MI quality (5.11±0.60) and concentration after MR (5.95±0.70) were similar throughout (all *P*>0.112). Estimation of MR difficulty (5.02±1.24) did not differ over blocks although the effect approached significance (F_5,50_ = 2.313, *P* = 0.057). According to the Kolmogorov-Smirnov test, the percentage of errors during MR was similar among participants (12.5±2.95%), indicative of homogeneity (z = −0.264, *P* = 0.791).

## Discussion

This study confirms a facilitatory interaction between rIPL and left M1 at rest in the majority of participants studied, and shows for the first time that this facilitation is withdrawn during both implicit and explicit imagery. The MEP facilitation observed at rest with a short ISI (6 ms) likely reflects a cortico-cortical interaction along a transcallosal pathway between rIPL and left M1 [21], [38], [39]. Interestingly, this MEP facilitation was abolished during both MI and MR, and reversed to suppression during MI when conditioning with an ISI of 12 ms indicating a potential role of rIPL in movement inhibition.

The MEP suppression during MI (12 ms ISI) may reflect an indirect inhibitory pathway between rIPL and left M1. Such a pathway may play a role in preventing movement execution during MI. In support of this idea, Schwoebel et al. [12] reported on a patient with bilateral parietal lobe damage who was unaware that he involuntarily executed hand movements when asked to imagine them. This suggests a role for IPL in preventing the execution of imagined movements, perhaps by reducing the net facilitation of corticomotor excitability during MI. Interestingly, the suppressive effect of rIPL stimulation was observed during MI and not MR, possibly because the risk of unwanted movement execution may be greater during explicit than implicit imagery. Although speculative, this difference might arise from the greater involvement of motor and sensorimotor cortical networks during MI [8] combined with the recruitment of an inhibitory pathway to avoid overt motor outflow.

It is worth considering how basal ganglia-thalamo-cortical pathways may be involved in suppressing unwanted movement during explicit imagery. During MI, and similar to movement execution, an efferent copy of descending commands may be sent to the input components of the basal ganglia (BG) [40]. These components are somatotopically organized, and relay signals via direct and indirect pathways through the BG and back to M1 via the ventrolateral thalamo-cortical pathway. This process focuses output from M1 by facilitating the desired movement representations, while inhibiting unwanted movement [41]. The rapid prevention of pre-planned movement in response to a stop cue may occur via a “hyper-direct” pathway [42] between the inferior frontal gyrus, the subthalamic nucleus and M1 [43]. Further research is needed to determine whether rIPL can modulate M1 excitability via this BG-thalamo-cortical pathway.

Another finding of interest was the lack of a long latency (12 ms) facilitatory effect of right parietal cortex stimulation on left M1 while participants were at rest, in contrast to the results of Koch et al. [21]. Differences in the stimulation site may explain these contrasting results. We calculated the coordinates of rIPL by averaging activation maxima previously reported in fMRI studies of MI and MR. Koch et al. [21] stimulated the caudal aspect of the right intraparietal sulcus, defined relative to the P4 position of the 10–20 EEG system. This site is located close to the angular gyrus in the inferior parietal lobule and the posterior part of the adjoining intraparietal sulcus [44]. In future, individualized neuro-navigation based on functional localization using fMRI could more clearly differentiate the effects of conditioning aspects of the parietal cortex [45].

The task-dependent nature of MEP disfacilitation from rIPL stimulation supports the idea that implicit imagery during MR and explicit imagery during MI involve independent neural processes. MR of a body part involves mental imagery as a ‘tool’ to manipulate the movement representation [8], whereas MI explicitly engages visual, sensorimotor and motor cortices to recreate visual and/or kinesthetic feedback [46]. MR and MI produced different time-dependent interactions between rIPL and left M1, at least as evidenced by the two ISIs examined in this study. MR disfacilitated M1 via a presumed direct pathway observed at rest, whereas MI disfacilitated M1 via a presumed indirect inhibitory pathway. These findings support the hypothesis that MR and MI are two distinct motor-related processes that engage different mechanisms of the inferior parietal lobe. While both tasks produce similar peaks of activation in rIPL, the location seems to be less variable during MI than during MR (see coordinates for rIPL location in MI [24], [25], [26], [27], [28], [29] and MR studies [8], [14], [30], [31], [32]). Though further research is needed to identify the pathway through which rIPL plays a role in inhibition during motor imagery, the present results give a first hint in that direction.

There were also unexpected results that might reflect potential limitations of the current study. One was that participants preferentially facilitated corticomotor excitability during either MI or MR, but not both. This should be taken into account in future studies when assessing neurophysiological effects of these two types of imagery. When all participants were pooled, NC MEP amplitudes were similar between MI, MR and rest conditions. The absence of facilitation during MI at the group level may be due to the varying individual pattern of modulation of NC MEP amplitude. A recent study showed a relationship between imagery ability and corticomotor excitability that could at least in part explain this heterogeneity [36]. Interestingly, the effects of either task on the rIPL-left M1 interaction were not affected by the participant-specific modulation of left M1 corticomotor excitability during MI or MR. This indicates that the processes responsible for facilitation of corticomotor excitability during MI are independent from the interaction between IPL and contralateral M1. While the IPL is involved in MI, its role does not appear to include facilitation of corticomotor excitability, and may instead involve disfacilitation. An attempt was made to optimise the timing of stimulation for each imagery task based on the known literature and our specific hypotheses. To some extent our selection of timings seems supported by the data. However, it is possible that modulatory effects between right IPL and left M1 occurred at other timings that were not tested, and these could be explored in future studies.

In conclusion, this study demonstrates a short-latency direct facilitation between rIPL-left M1 at rest, and shows for the first time that this facilitation is withdrawn during both implicit and explicit imagery, independently of the facilitation of corticomotor excitability by either form of imagery. During explicit motor imagery a long-latency indirect suppression occurs between rIPL-left M1 and may contribute to the prevention of unwanted movement.
